# Unraveling the active site cover of coproheme decarboxylase from *Listeria monocytogenes*

**DOI:** 10.1111/febs.70575

**Published:** 2026-05-06

**Authors:** Nikolaus Falb, Dominik Hörtnagel, Chris Oostenbrink, Paul G. Furtmüller, Stefan Hofbauer

**Affiliations:** ^1^ Department of Natural Sciences and Sustainable Resources, BOKU University, Institute of Biochemistry Vienna Austria; ^2^ Department of Natural Sciences and Sustainable Resources, BOKU University, Institute of Molecular Modeling and Simulation Vienna Austria

**Keywords:** heme biosynthesis, MD simulations, molecular enzymology, spectroscopy, structure–function relationships

## Abstract

Coproheme decarboxylase (ChdC) is the terminal enzyme in Gram‐positive heme *b* biosynthesis, an enzyme holding a special importance given its unique structure–function relationship and its necessity for bacterial survival. In the past, the enzyme has been shown to perform a double decarboxylation of two propionate groups on coproheme (Fe(III)‐coproporphyrin III), subsequently converting it to heme *b* (iron‐protoporphyrin IX). Notably, the active site of ChdCs is universally covered by a flexible loop. Its importance has not been fully studied but given its position it is assumed to provide steric hinderance for substrates or potential inhibitors to pass. This study aims to investigate its physiological role by introducing a histidine‐to‐alanine mutation located on the loop of ChdC from *Listeria monocytogenes*. Molecular dynamics simulation shows an increased flexibility of the structural element in the mutant. As a consequence, various kinetic studies at steady‐ and presteady‐state conditions suggest coproheme binding and active site accessibility are improved. Using simulations and X‐ray crystallography, we show evidence that the loop is originally stabilized by a hydrogen bond between S116 and the mutated H117. The higher accessibility also results in a higher susceptibility to damage from oxidative cosubstrates like H_2_O_2_, suggesting the loop in its wild‐type conformation plays a key biological role in regulating the transfer of cosubstrates towards the main substrate coproheme.

## Graphical abstract

During heme biosynthesis in Gram‐positive bacteria, coproheme decarboxylase (ChdC) catalyzes the conversion of four‐propionate substrate coproheme to the two propionate product heme *b*. Its active site is universally covered by a flexible linking loop. This study identifies an important histidine residue, which stabilizes the loop in a ChdC homolog. A point mutation increased active site accessibility and subsequently increased oxidative damage caused by cosubstrates. Our results indicate a protective function of the loop.

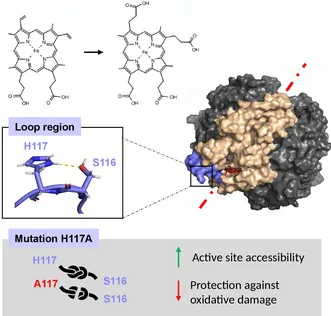

**Abbreviations**
5‐ALA: 5‐aminolevulinic acid
CCP4: Collaborative Computational Project No. 4
CD: circular dichroism
CgoN: alternative coproporphyrinogen III oxidase
CgoX: coproporphyrinogen III oxidase
ChdC: coproheme decarboxylase
CPD: coproporphyrin‐dependent
CpfC: coproporphyrin ferrochelatase
ESRF: European Synchrotron Radiation Facility
FMN: flavin mononucleotide
H117A
: histidine 117 replaced with alanine
HEPES: 4‐(2‐hydroxyethyl)‐1‐piperazineethanesulfonic acid
HPLC: High‐performance liquid chromatography
HQ‐H_2_O: High‐quality water
HRV3C: human rhinovirus 3C (protease)
IPTG: isopropyl β‐*d*‐1‐thiogalactopyranoside
*K*_D_
: dissociation constant
*k*_off_
: dissociation rate constant of cyanide
*k*_on_
: bimolecular association rate constant of cyanide binding
LC–MS: liquid chromatography‐mass spectrometry
LC‐TOFMS: liquid chromatography‐time of flight mass spectrometry
MALS: multi‐angle light scattering
MD: molecular dynamics simulations
MMD: monovinyl, monopropionate deuteroheme
MS: mass spectrometry
NaCl: sodium chloride
OD600: optical density at 600 nm
PAA: peroxyacetic acid
PBS: phosphate buffered saline
PDB: protein data bank
PEG: polyethylene glycol
PPD: protoporphyrin dependent
RMSD: root mean square deviation
RMSF: root mean square fluctuation
SEC: size exclusion chromatography
UPLC: ultra‐performance liquid chromatography
WT: wild‐type

## Introduction

Coproheme decarboxylase (ChdC) is the terminal enzyme in the Gram‐positive heme biosynthesis pathway CPD (coproporphyrin‐dependent pathway) [1, 2]. A loss or dysfunction of ChdC has shown to be associated with heme *b* deficiency and severe phenotypes across different organisms and conditions [3, 4, 5]. ChdC is a valuable target for enzymatic research, as it is unique to Gram‐positive bacteria, a group containing a plethora of pathogenic organisms and might prove to be a suitable target for future antibiotics [6].

The CPD pathway for heme biosynthesis consists of the final three enzymatic steps to heme *b*. Earlier steps of heme biosynthesis initiate with universal precursor 5‐aminolevulinic acid (5‐ALA) and result in the emergence of coproporphyrinogen III, from which point onwards, biosynthesis either follows the protoporphyrin dependent pathway (PPD) in eukaryotes and Gram‐negative bacterial species or deviates into the CPD pathway [1, 2].

Figure 1 depicts the initial six‐electron oxidation from coproporphyrinogen III to coproporphyrin III, mediated by coproporphyrinogen III oxidase (CgoX) or anaerobic coproporphyrinogen III oxidase (CgoN) [7, 8]. The next step is the insertion of ferrous iron into the porphyrin skeleton by coproporphyrin III ferrochelatase (CpfC) and finally the oxidative decarboxylation of two propionate groups on coproheme (iron coproporphyrin III) to yield heme *b*, as mediated by ChdC [8].

**Fig. 1:**
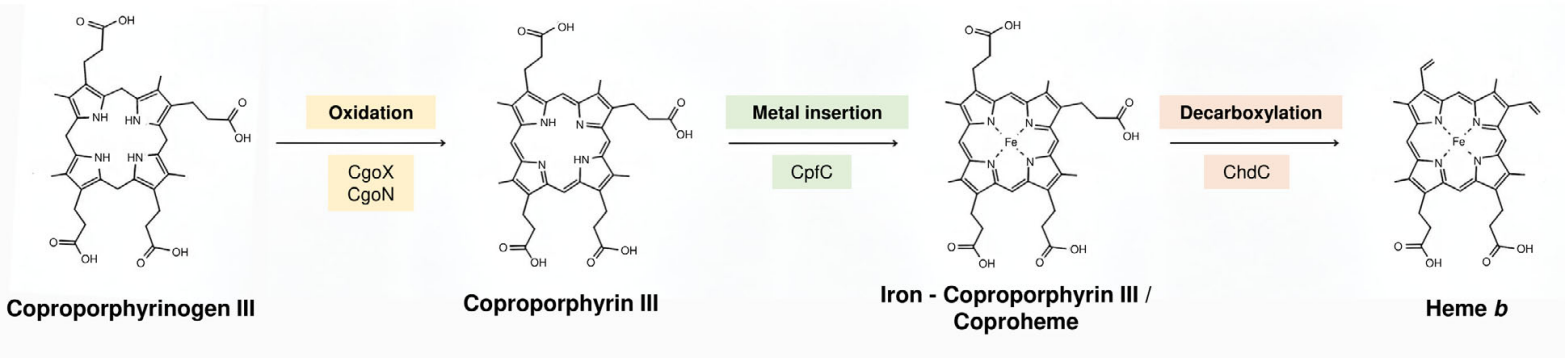
Steps of the CPD pathway for heme biosynthesis, starting from coproporphyrinogen III.

The sequential decarboxylation of the propionate groups involves a three‐propionate intermediate (monovinyl monopropionate deuteroheme (MMD)), which subsequently reorients and is decarboxylated itself [9, 10].

$$\text{(I) Coproheme}+H_{2}O_{2}\to \mathrm{MMD}+2\;H_{2}O+{\mathrm{CO}}_{2}$$

$$\mathrm{(II)}\;\mathrm{MMD}+H_{2}O_{2}\to \text{Heme}\;b+2\;H_{2}O+{\mathrm{CO}}_{2}$$

Notably, the reaction requires an oxidative cofactor to stimulate the formation of an oxygenated iron‐porphyrin radical species (Compound I) and to take up emerging electrons during the oxidative decarboxylation process. Known substrates are H_2_O_2_, which emerges during earlier steps of the aerobic CPD pathway [8], the less aggressive FMN (flavin mononucleotide) under aerobic and anaerobic conditions [1, 3] and other known peroxidase substrates like ${\mathrm{ClO}}_{2}^{-}$ (chlorite) or PAA (peracetic acid) [11, 12].

As iron‐porphyrin cofactors such as coproheme and MMD are somewhat susceptible to oxidative damage by oxidative substrates, resulting in loss of absorbance (bleaching) [13, 14], ChdC's active site is covered by a flexible loop connecting the two ferredoxin‐like domains of the enzyme subunit [9, 10]. This loop varies in length depending on the origin of the enzyme. Figure 2A,B show crystal structures of ChdC from *Listeria monocytogenes* (*Lm*ChdC) and from *Corynebacterium diphtheriae* (*Cd*ChdC) with their flexible elements marked in yellow. It has been shown that this loop is elongated for *Firmicute* ChdCs, like *Lm*ChdC, in comparison to actinobacterial ChdCs, like *Cd*ChdC. In *Cd*ChdC, this loop has catalytical implications, enabling a histidine residue (H118) to participate in the reaction as a distal base [9]. This histidine is conserved and relevant across the actinobacterial clade [10].

**Fig. 2:**
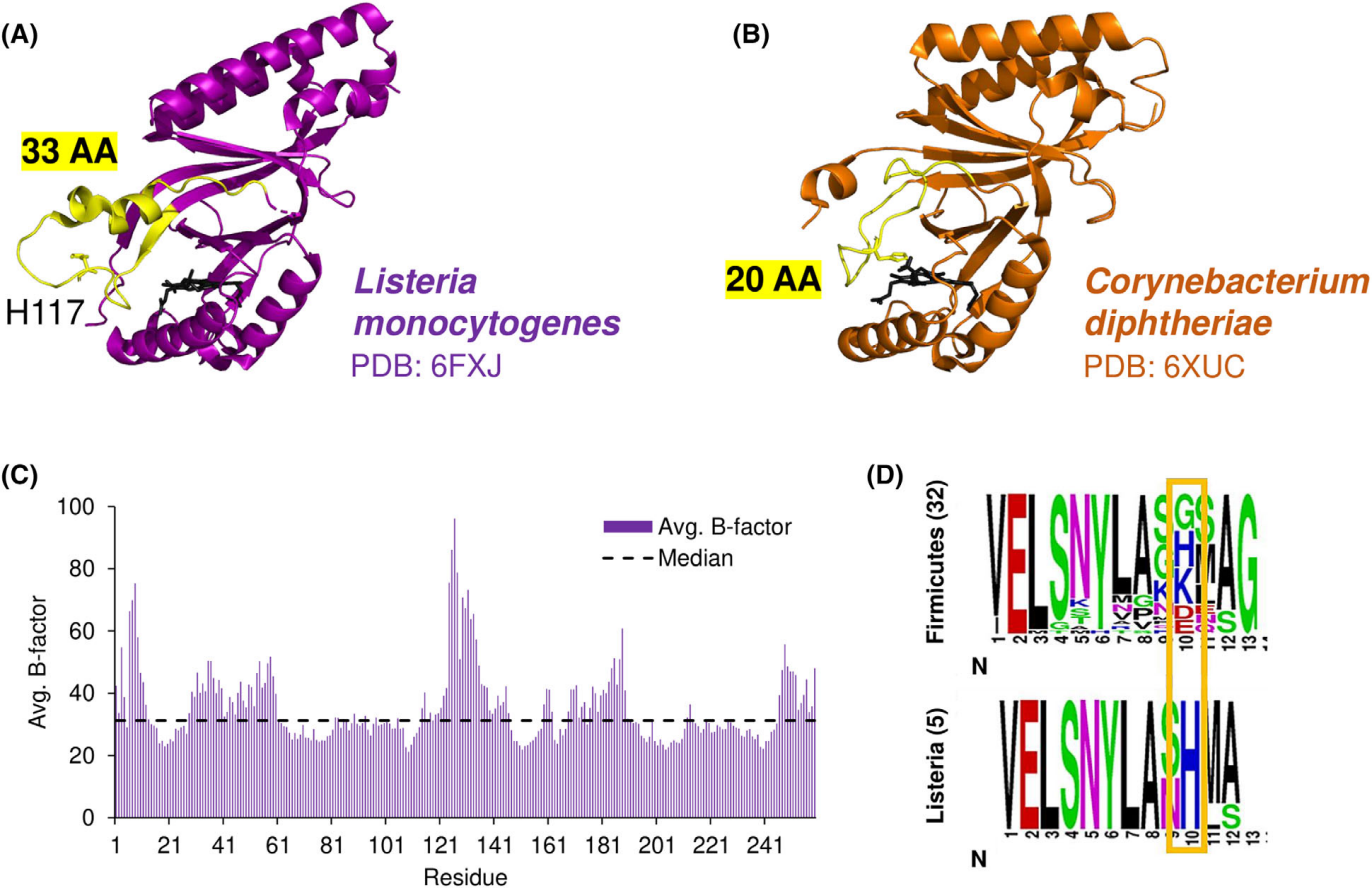
Analysis of ChdC subunits of different origins regarding structure and sequence. (A) Crystal structure of coproheme bound ChdC from Listeria monocytogenes (PDB‐ID: 6FXJ) depicted in pymol. The overall structure is shown in pink with the flexible loop covering the active site shown in yellow. Further highlighted in stick depiction are the coproheme (black) and histidine on position 117. (B) Crystal structure of coproheme bound ChdC from Corynebacterium diphtheriae (PDB‐ID: 6XUC) in pymol. The overall structure is shown in orange with the flexible loop covering the active site shown in yellow. Further highlighted in stick depiction are the coproheme (black) and histidine on position 118. (C) Average B‐factor of all deposited ChdC structures (September 2025) in the PDB, assigned to their position in the consensus sequence. Median is highlighted by a dashed line. (D) First 13 amino acids of the flexible linking loop of 32 Firmicutes or 5 Listeria ChdCs, derived from a multiple sequence alignment and depicted by Berkeley WebLogo.

B‐factor analysis of all available ChdC crystal structures (September 2025) shows that the loop around residues 120–145 in the consensus sequence has an increased B‐factor when resolved (Fig. 2C), highlighting its flexibility and its conservation throughout the ChdC cosmos. Often the loop is not resolved at all: For example, in the crystal structure of *Lm*ChdC (PDB: 6FXJ) it is only resolved in two out of five subunits, stabilized in this position due to crystal packing contacts [12].

Given loop dynamics and sequence–function relationship is better studied in actinobacterial ChdCs [9, 10, 15], we continued with *Lm*ChdC as a *Firmicute* model system. Notably, multiple sequence alignments show a histidine residue conserved in ChdCs from the taxon *Listeria* (position H117 in *Lm*ChdC) (Fig. 2D). This histidine was exchanged with an alanine residue and its impact on enzyme activity and active site accessibility was investigated.

Subsequently we show that the histidine has a different relevance for *Lm*ChdC than H118 has for *Cd*ChdC: Its loss causes a higher degree of loop flexibility and substrate accessibility. The latter is improved, which is in turn causing a higher susceptibility to oxidative damage caused by the cosubstrate. On the other hand, we did not observe a significant role of the loop‐histidine as a distal base for hydrogen peroxide deprotonation, as for the actinobacterial ChdCs.

## Results

### Molecular dynamics simulation of *Lm*ChdC WT and H117A mutant

Figure 3 summarizes the results of the 50 ns simulations of the wild‐type and mutant: The RMSF analysis (Fig. 3A) shows increased backbone RMSF values across nearly all atoms of the defined loop regions in the H117A mutant compared to the WT simulation. Notably, the fourth loop exhibits elevated RMSF values even in the WT relative to the other loops. A similar increase in RMSF is observed for this loop in the mutant simulation.

**Fig. 3:**
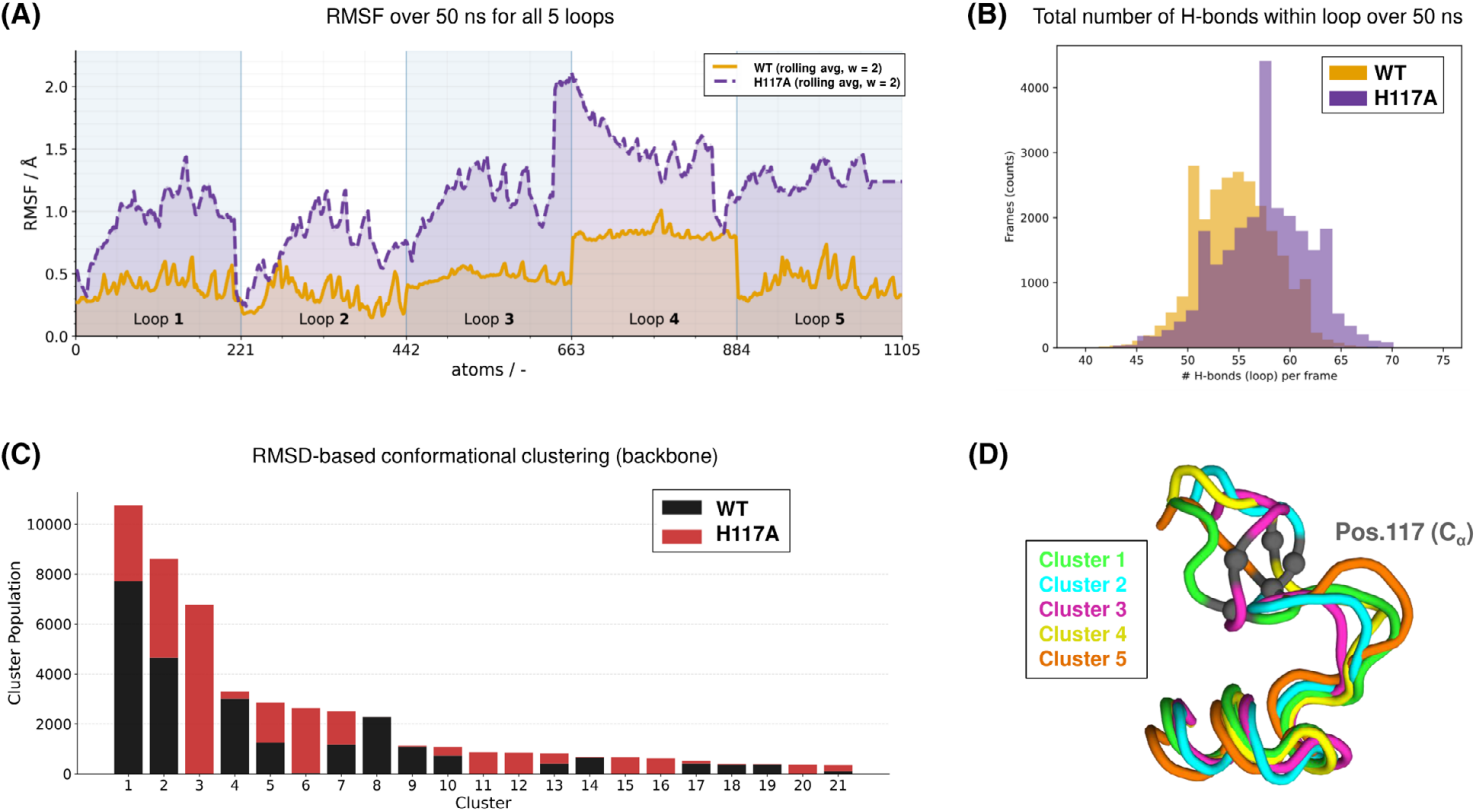
Results of molecular dynamics simulation on *Lm*ChdC WT and H117A. (A) RMSF analysis for the backbone atoms of the loop regions of the pentameric enzyme over 50 ns of simulation time. WT is depicted in yellow and H117A in a violet dashed line. (B) Distribution of all hydrogen bonds per frame within the loop region. WT is shown in yellow, and H117A in violet. (C) RMSD‐based conformational clustering showing the population of clusters sampled during the WT (black) and H117A (red) simulations. (D) Representative structures of the five most populated clusters based on the combined (WT + H117A) population with the Cα atoms of residue 117 highlighted as gray spheres.

Analysis of backbone hydrogen bonds (Fig. 3B) shows that the H117A mutant samples a broader distribution of hydrogen bond counts per frame compared to the WT trajectory. The mutant distribution is slightly shifted toward higher hydrogen bond counts, indicating the formation of additional transient backbone hydrogen bonds. This broader distribution suggests a more dynamic hydrogen bonding network within the loop regions of the mutant. Figure S1 provides additional insight into the nature of backbone hydrogen bonds and shows that additional backbone hydrogen bond contacts appear in the mutant trajectory, which may relate to the occurrence of short‐lived alternative conformations sampled by H117A during the simulation.

Additional insight is provided by RMSD‐based conformational clustering (Fig. 3C), which reveals a larger number of distinct conformational states sampled by the mutant trajectory. Several clusters (3, 6, 11, 12, 15, 16, and 20) are populated exclusively by loop conformations observed in the H117A simulation, whereas clusters 4, 8, 9, 14, 17, 18, and 19 are predominantly populated by conformations observed for the WT. The remaining clusters are shared between both simulations. When examining the five most populated clusters, cluster 1 contains the largest population and is composed of more than 50% WT frames. Cluster 2 is populated approximately equally by WT and mutant frames, whereas cluster 3 is populated exclusively by the H117A simulation. Cluster 4 is about half the size of cluster 1 and consists predominantly of WT frames, while cluster 5 contains a similar number of frames from both simulations. The central representative structures of clusters 1–5 are presented in Fig. 3D.

Taken together, these results indicate that the H117A mutation leads to increased loop flexibility and a broader conformational ensemble relative to the WT simulation. This is reflected by elevated RMSF values, reduced backbone hydrogen bond occurrence, and a more diverse clustering profile.

### Size control by HPLC

Analytical size exclusion HPLC on fully purified and − 80 °C stored protein was performed with MALS and UV (280 nm) detection (Fig. S2). Elution behavior shows that WT and mutant both elute at an equivalent retention time of 17 min in a sharp singlet peak resembling SEC purification and verifying a lack of aggregation and high storage stability. MALS detection reveals a *M*_W_ of slightly above 150 kDa for both, which aligns nicely with a homopentameric *Lm*CdhC.

### CD spectroscopy of apo‐enzyme

To further investigate secondary structure differences in solution, CD spectroscopy was performed and resulted in a visible difference between WT and H117A (Fig. 4). A useful metric to quantify differences for *Lm*ChdC is the ratio of ellipticity between 208 and 222 nm, as shown by Milazzo *et al*., [16]. In direct comparison, this ratio is elevated for the mutant, which highlights a significant change.

**Fig. 4:**
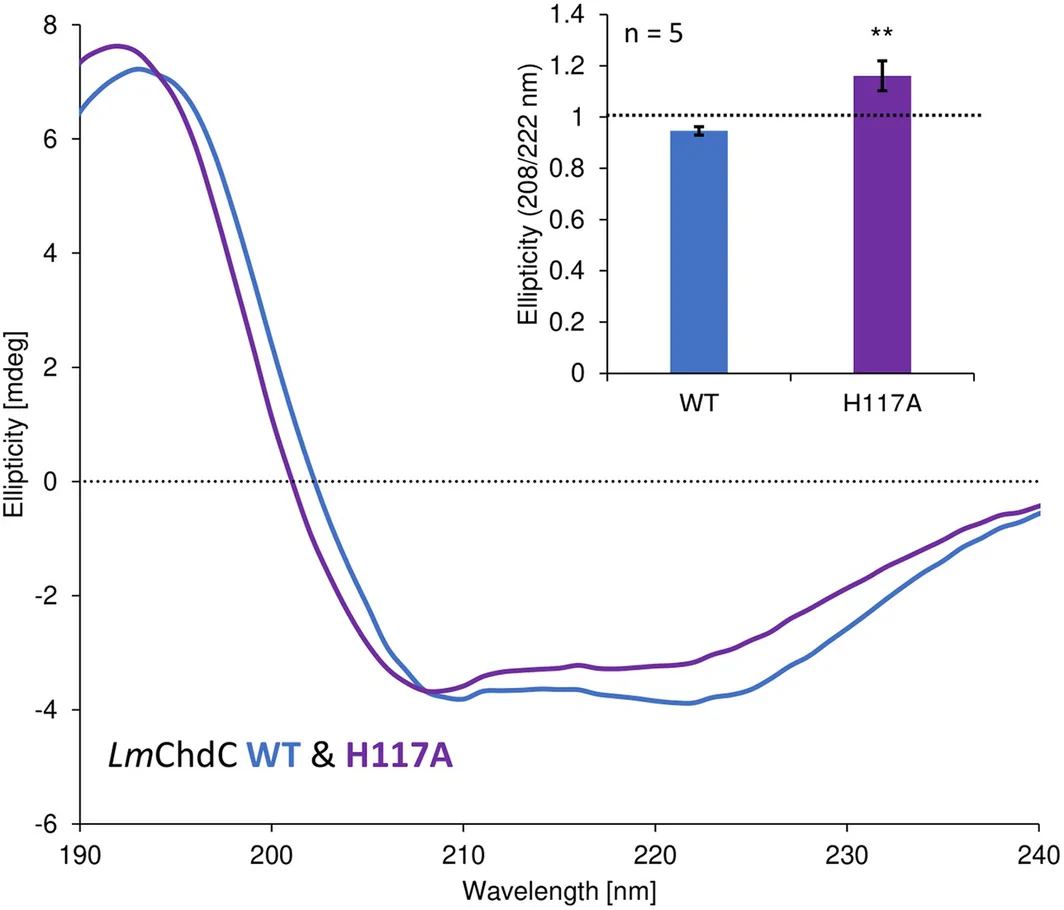
CD spectra of *Lm*ChdC WT and H117A from 190 to 240 nm. WT (blue) and H117A (violet) are depicted in overlay. Spectra represent average of 5 biological replicates. Inset shows the ellipticity ratio between 208 and 222 nm for WT and H117A. Bar plots represent average with standard deviations; a Mann–Whitney rank sum test resulted in a significant difference (*P* = 0.008), ‘**’refers to a significance level of *P* < 0.01.

Upshifts in the 210–230 nm area, as seen for H117A in comparison to the WT, are associated with an increase in disordered random coil as a secondary structural element [17]. Alongside the previously presented simulations, this paints the picture of a more disordered mutant, associated with the loss of secondary structure, especially in the already fragile loop area.

### Coproheme binding to *Lm*ChdC WT and H117A mutant

Initially binding of coproheme was verified by a steady‐state experiment, in which coproheme was titrated with increasing amounts of apo‐protein until a saturation was reached (Fig. S3). Absorbance characteristics of the Soret peaks of WT and H117A are highly similar and saturation is reached for both at a 1 : 1 molar ratio of protein to coproheme, verifying the stoichiometry of one cofactor molecule bound to one monomer.

The binding of coproheme was recorded in a stopped‐flow experiment which showed that an intermediate state formed, when the WT protein bound the porphyrin after 0.2–0.5 s (orange trace in Fig. 5A,B). The estimated rate constant for the appearance of the intermediate was *k* = 3.7 × 10^7^ M^−1^·s^−1^. A second slower step (*k* = 1.1 M^−1^·s^−1^) occurred, leading to the loss of the intermediate state and to the formation of the final bound spectrum (blue in Fig. 5A,B). This intermediate state is clearly distinguishable to the final state, since the 375 nm shoulder is not visible yet (Fig. 5A). A 375 nm shoulder is a typical absorbance band for coproheme bound ChdCs as described in previous publications and is visible in Fig. S3 [18, 19].

**Fig. 5:**
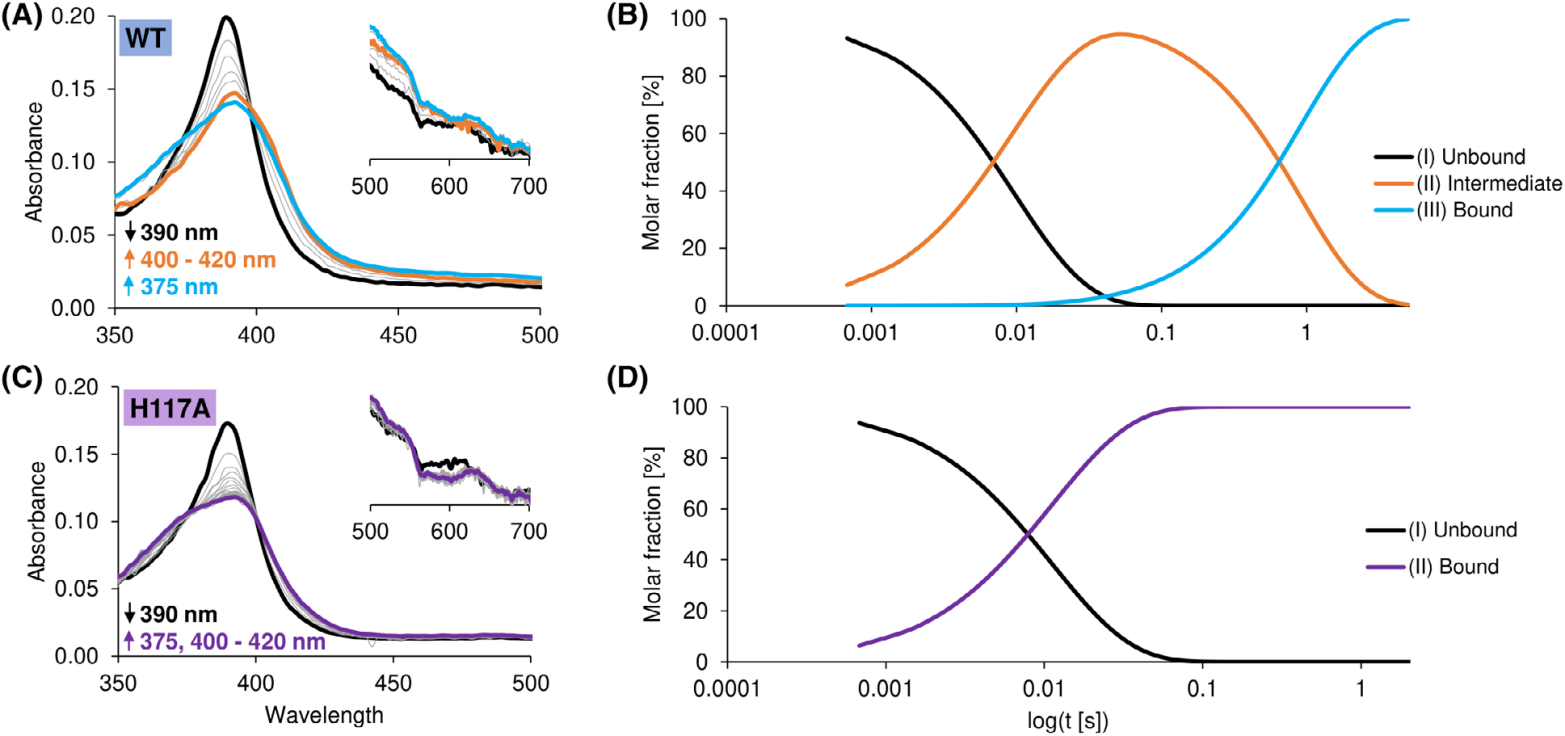
Kinetics of coproheme binding to *Lm*ChdC WT and H117A by stopped‐flow spectroscopy. (A) Spectral shift from unbound coproheme (black) mixed with the WT, to a visible intermediate state (orange), to the bound state (blue) over the course of 5 s. (B) Molar fractions of the different spectral species emerging in the WT experiment, calculated by *Pro‐Kineticist* and over the course of 5 s, with a logarithmic time scale to visualize the rapid shift. (C) Spectral shift from unbound coproheme (black) mixed with H117A, to the bound state (violet) over the course of 5 s. (D) Molar fractions of the different spectral species emerging in the H117A experiment, calculated by *Pro‐Kineticist* and over the course of 5 s, with a logarithmic time scale to visualize the rapid shift.

In contrast in the H117A variant, no spectral signatures indicative of an intermediate state are present. Coproheme binds in a monophasic behavior with spectral transitions exhibiting clear isosbestic points at 375 and 400 nm. The final bound spectrum, verified in Fig. S3, is reached directly with an estimated rate of *k* = 3.2 × 10^7^ M^−1^·s^−1^. (Fig. 5C) The bound state (violet) is fully established at approximately the same time the WT takes to reach its intermediate state (Fig. 5D).

### Enzyme activity by LC–MS

Figure 6 shows different levels of coproheme conversion by WT and H117A with H_2_O_2_, PAA, and ${\mathrm{ClO}}_{2}^{-}$. Both enzymes reach full conversion of coproheme to heme *b* at a 15‐fold substrate excess and have only negligible conversion with no oxidant present. Intermediate steps slightly vary between mutant and WT. However, the most notable difference is a strong loss of conversion of the mutant with H_2_O_2_ between two‐ and fivefold excess. This indicates the mutant is less efficient when utilizing H_2_O_2_.

**Fig. 6:**
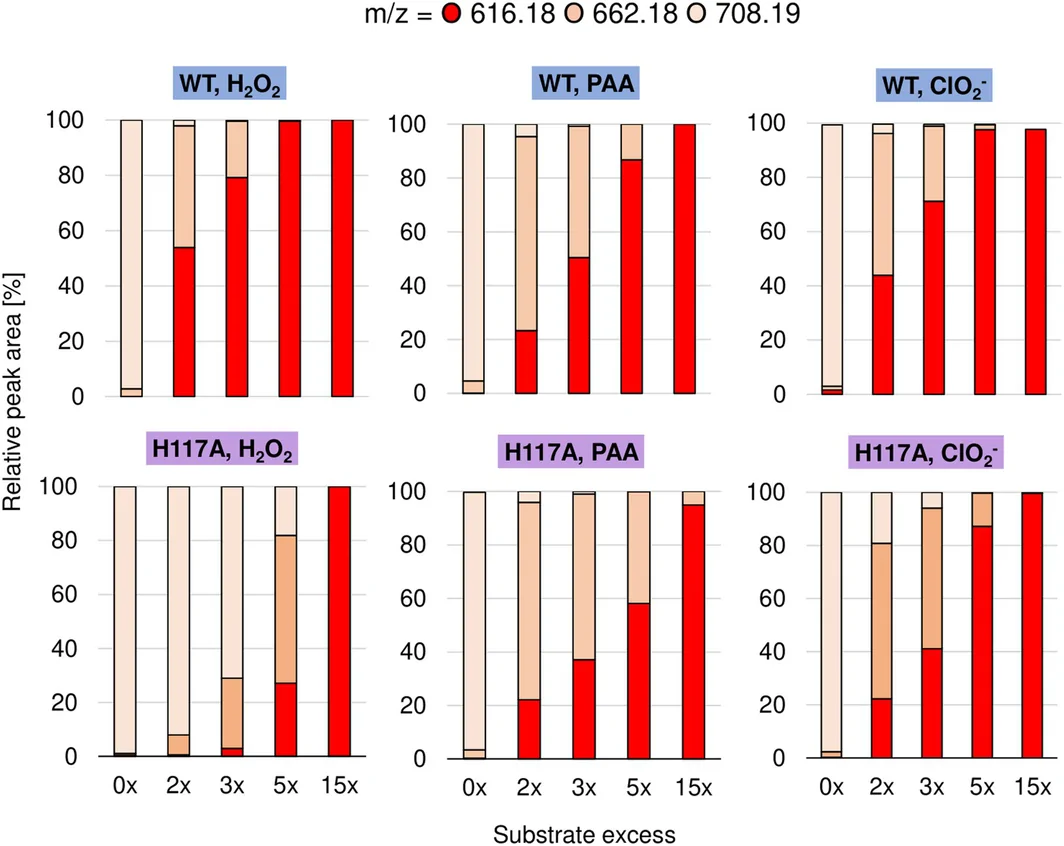
Peak area fractions of coproheme (708.19 Da, light), intermediate MMD (662.18 Da, flesh) and heme *b* (616.18 Da, red) of *Lm*ChdC WT and H117A incubated with different molar excesses of cosubstrates H_2_O_2_, PAA or ${\mathrm{ClO}}_{2}^{-}$ (*n* ≥ 3).

### Enzyme activity by spectroscopy

To investigate this discrepancy, a H_2_O_2_ titration under steady‐state conditions was followed by UV–vis spectroscopy (Fig. 7). When comparing the spectral shift with rising H_2_O_2_ to enzyme ratio, the WT enzyme (Fig. 7A) undergoes a clear shift in its Soret peak to 410 nm (heme *b* bound *Lm*ChdC, see [20]). An isosbestic point is visible at 400 nm. Afterwards, a spectral decline across the whole Soret peak is visible, most likely related to heme *b* bleaching, a phenomenon typical for heme enzymes exposed to high levels of oxidant [13, 14]. After approximately threefold excess of H_2_O_2_, coproheme is completely converted into heme *b* (Fig. 7B).

**Fig. 7:**
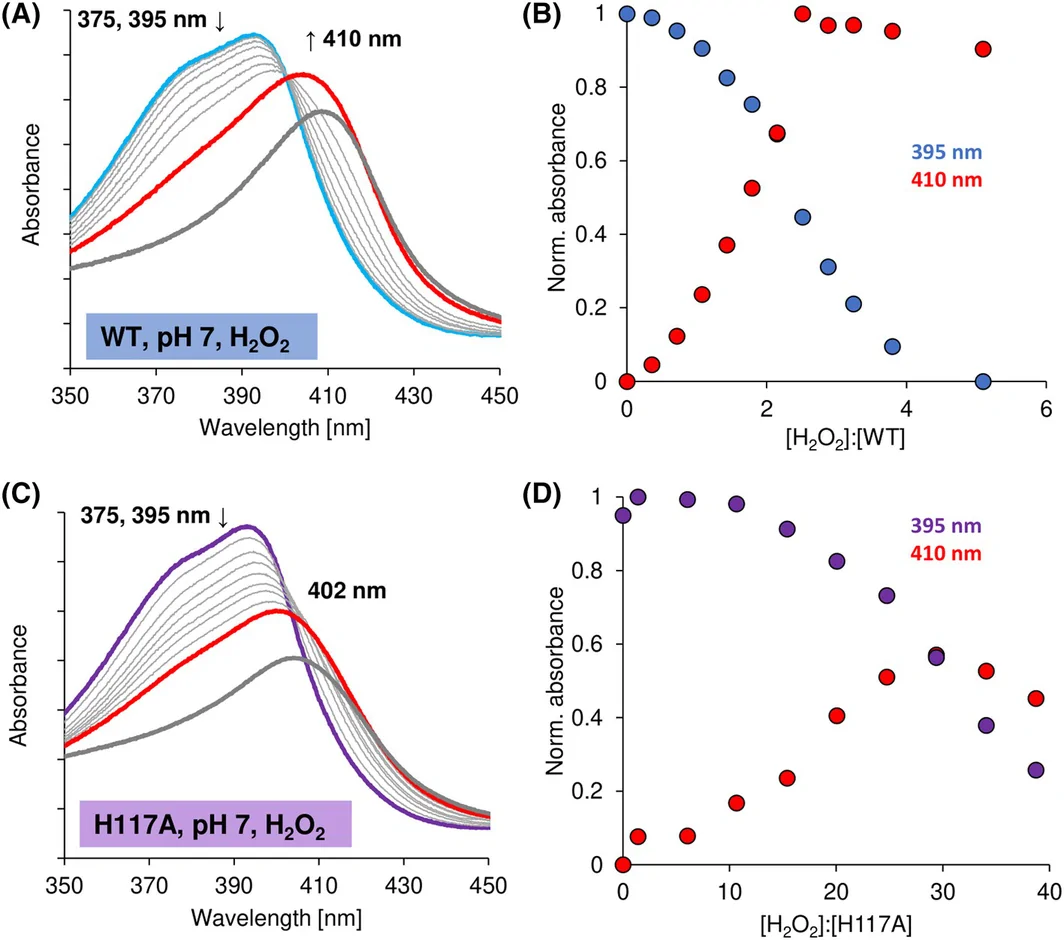
Titration of coproheme bound *Lm*ChdC WT and H117A with H_2_O_2_, followed by photometry. (A). Spectral shift during titration of coproheme bound WT with cosubstrate H_2_O_2_, from substrate bound (blue) to product bound (red) to bleached state (gray) (*n* ≥ 3). (B) Normalized absorbance at 395 nm (coproheme associated, blue) and 410 nm (heme *b* associated, red) with increasing molar H_2_O_2_ to enzyme ratio. (C) Spectral shift during titration of coproheme bound H117A with cosubstrate H_2_O_2_, from substrate bound (violet) to product bound (red) to bleached state (gray). (D) Normalized absorbance at 395 nm (violet) and 410 nm (red) with increasing molar H_2_O_2_ to enzyme ratio. The absorbance at 410 nm was normalized to the maximal absorbance at 410 nm from the WT data (shown in Fig. 4A).

The bleaching effect is more pronounced for H117A, as can be seen from the fact that the peak at 410 nm does not appear before spectral decline dominates (Fig. 7C). A lack of a clear isosbestic point and no clear formation of a pronounced 410 nm peak indicates a high susceptibility to oxidative damage for the mutant. When attempting to track heme *b* formation at 410 nm (Fig. 7D), it noticeably takes higher H_2_O_2_ levels to see a rise in absorbance. With more H_2_O_2_ added, absorbance rises slowly, with 395 nm absorbance declining correspondingly, indicating heme *b* is formed. After 30‐fold molar excess of H_2_O_2_, heme *b* formation halts at about 60% of the absorbance at 410 nm the WT can reach, indicating bleaching of heme *b* dominates and might be more strongly pronounced than the bleaching of coproheme.

### Cyanide binding characterized by thermodynamic and pre‐steady‐state studies

Cyanide binding studies help to understand substrate/ligand channeling toward the active site. On the one hand, information on steric constraints is obtained and the other hand cyanide binding experiments also yield information of the deprotonation potential of the active site, which is needed for hydrogen peroxide activation.

Figure 8A–F show the spectral shift that *Lm*ChdC undergoes when it is titrated with increasing concentrations of cyanide. Both, WT (Fig. 8A,B) and H117A (Fig. 8C,D) show a spectral shift of the Soret double peak (375, 395 nm) toward a singlet 412 nm peak resembling a low‐spin iron‐porphyrin species. Higher wavelength regions indicate the same behavior with a loss of absorbance at 500 and 635 nm and a gain of absorbance in a broader 530–600 nm area.

**Fig. 8:**
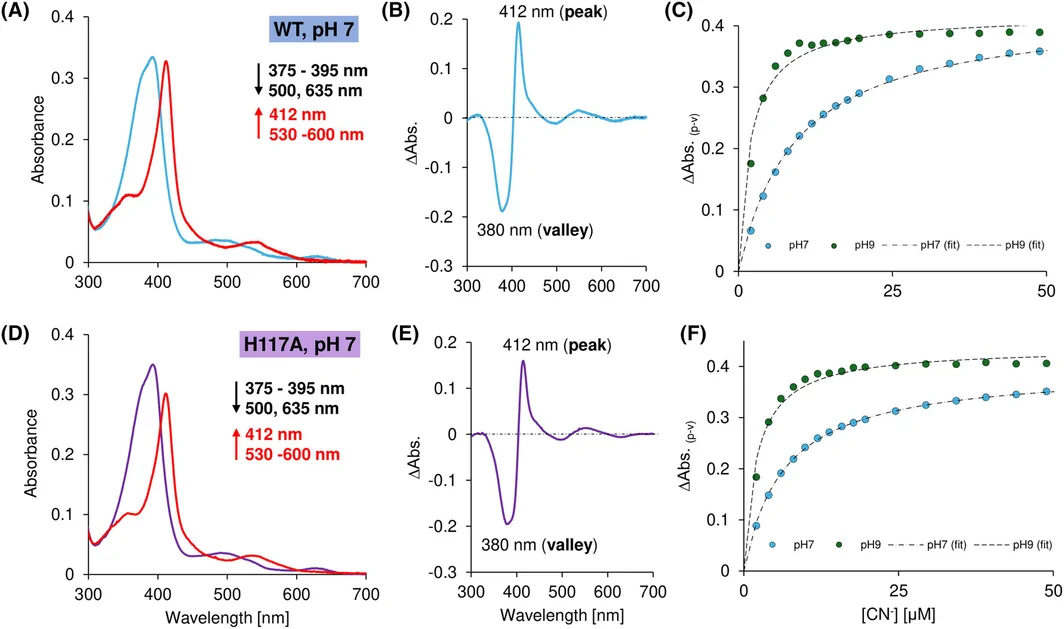
Spectral shifts observed during cyanide titration (A) Spectral shift of WT from resting state (blue) to ligand bound state (red) (*n* ≥ 3). (B) Associated differential spectrum highlighting absorbance change minimum (valley) and maximum (peak) for the WT. (C) Combined absorbance change at peak and valley plotted against different cyanide concentrations and at pH 7 and pH 9, for the WT. Hyperbolic fit shown as dashed line (D) Spectral shift of H117A from resting state (violet) to ligand bound state (red). (E) Associated differential spectrum for H117A. (F) Combined absorbance changes at peak and valley plotted against different cyanide concentrations and at pH 7 and pH 9, for H117A.

When subtracting the initial spectrum from the final spectrum after cyanide addition, it yields a difference spectrum for each sample (shown for the highest cyanide concentration in Fig. 8B,D). Here the wavelength, where the highest amount of absorbance is lost, is 380 nm (valley, v) and the wavelength where most absorbance is gained is 412 nm (peak, p). Figure 8C,E show the absorbance difference at p and v, plotted against the cyanide concentration for pH 7 and pH 9. The data are fitted hyperbolically, as described in the Method section. The fit reveals the binding parameter *K*_D_ to be increased for WT and mutant at pH 9 compared to pH 7 (Table 1). While the *K*_D_ at pH 9 is the same for both proteins, *K*_D_ at pH 7 is slightly decreased for H117A in comparison to the WT.

**Table 1:** Thermodynamic and kinetic parameters of cyanide complex formation of the *Lm*ChdC WT and H117A mutant.

Mutant	pH	*K*_D_ [μm] (by titration)	*k*_on_ [μm ^−1^·s^−1^]	*k*_off_ [s^−1^] (calculated)
WT	7	9.41 ± 0.17	0.0213	0.200 ± 0.004
	9	1.92 ± 0.22	0.302	0.580 ± 0.066
H117A	7	6.46 ± 0.06	0.120	0.775 ± 0.007
	9	2.05 ± 0.19	0.161	0.330 ± 0.031

To determine the on‐rate *k*_on_, cyanide binding was determined in the presteady‐state by tracking absorbance at 412 nm. *k*_off_ values, given their tendency to produce a high error rate in presteady‐state experiments. Instead, they were calculated with multiplying *k*_on_ with *K*_D_ values from the titrations.

The first difference between WT and the H117A variant is the curve fitting: While the WT requires a double exponential fit at pH 7 and a triple exponential fit at pH 9, a single exponential fit can be applied to H117A (Fig. 9A). *k*_obs,2_ which is only present for the WT and *k*_obs,3_, which is only present for the WT at pH 9, are not correlated with the cyanide concentration as shown in Fig. 9C,D. This indicates concentration‐independent structural changes within the active site after initial cyanide binding. These cyanide concentration independent changes in absorbance are presumably due to the formation of final interactions between the cyanide bound coproheme and active site residues and possibly also reflect the steric situation along the substrate/ligand access channel. The variant with the smaller alanine residue on the flexible loop does not interfere with the incoming cyanide compared to the WT, which is reflected in the multiphasic binding behavior. *k*_obs,1_ correlates linearly with the cyanide concentration for both samples at both pH values (Fig. 9B) and derives from the initial parts of the time traces, thereby fitting the major part of the absorbance change.

**Fig. 9:**
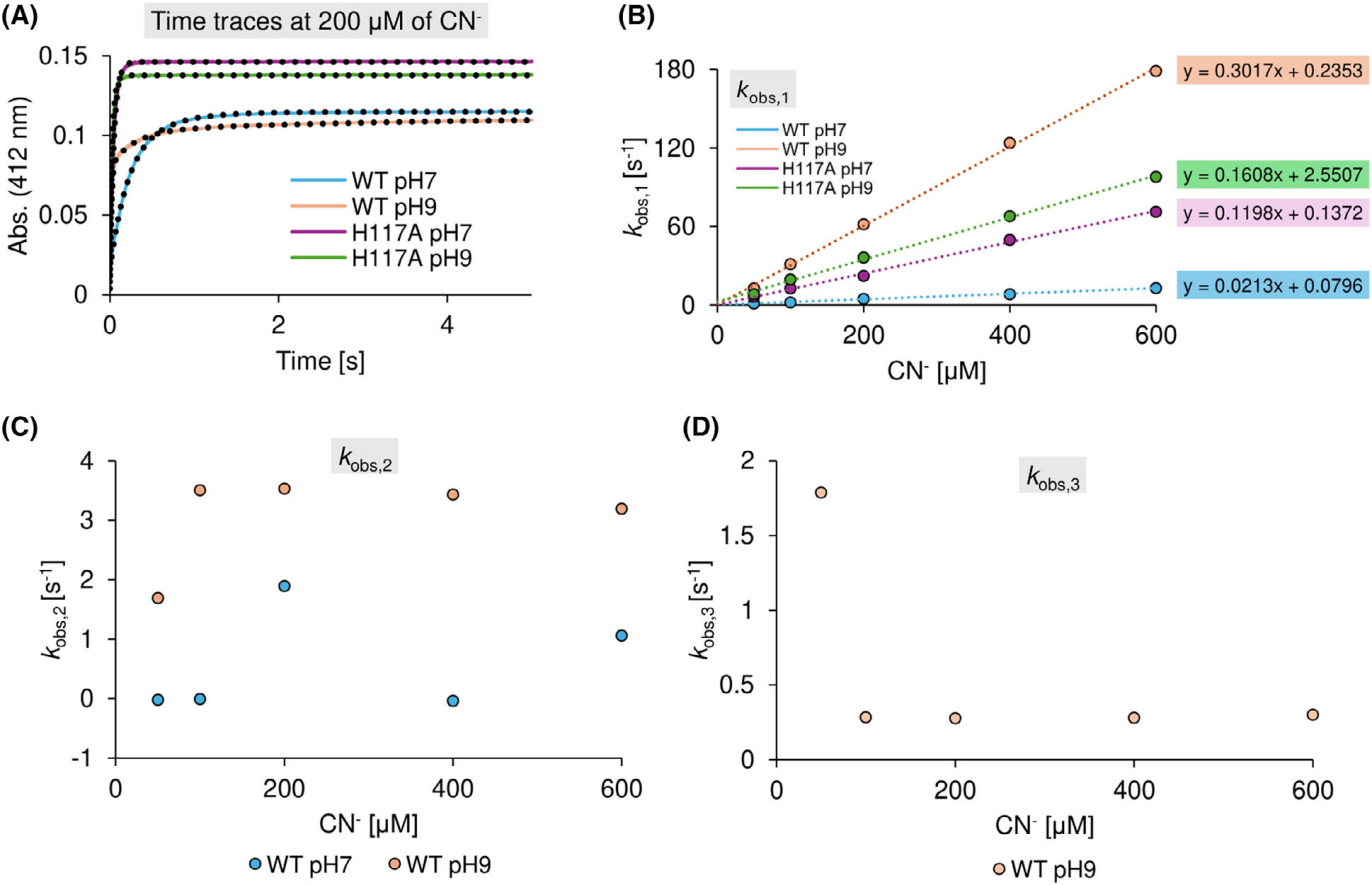
Presteady‐state kinetics of cyanide binding. Different proteins and pH values are color coded as: WT at pH 7 (blue), WT at pH 9 (orange), H117A at pH 7 (violet), H117A at pH 9 (green). (A) Time traces of absorbance at 412 nm over the course of 10 s and at 200 μm of cyanide (*n* ≥ 3). Exponential fits applied were overlaid as black dotted graphs. (B) *k*_obs,1_ against cyanide concentrations, linear regression shown as dotted lines, associated formulas are added in the corresponding color. (C) *k*_obs,2_ against cyanide concentrations, for measurements where it is applied. (D) *k*_obs,3_ against cyanide concentrations, for measurements where it is applied.

Based on the linear fit, *k*_on_ was calculated and can be reviewed in Table 1. Notably, *k*_on_ at pH 7 is about fivefold decreased for the WT relative to H117A. This is aligning with the results from the titration and indicates increased cofactor accessibility due to the mutation. At pH 9, WT's *k*_on_ increases 15‐fold in comparison to pH 7, while H117A barely increases in on‐rate. This increase in on‐rate at alkaline pH is expected if only moderate deprotonation of the cyanide by the histidine is happening. This is due to the higher binding rate of deprotonated cyanide, explained by its p*K*_a_‐value of 9.2 at room temperature. A highly efficient Lewis base to deprotonate the incoming HCN would manage to yield an even more similar on‐rate at pH 7. In case H117 would not act as a Lewis base for deprotonation at all, a 100‐fold increase of the on‐rate at pH 9 compared to pH 7 would be expected. This deprotonation is of course also relevant for the oxidative substrate hydrogen peroxide that needs to be activated by deprotonation. *k*_off_ values are of similar range throughout all conditions, suggesting the change in on‐rate is the main determining factor for the dissociation constant.

### Comparison of the crystal structure of *Lm*ChdC and the H117A mutant

Data collection and model parameters for the crystal structure of *Lm*ChdC H117A (PDB‐ID: 9T54) are depicted in Table 2. Figure 10 shows the structure of WT *Lm*ChdC (PDB: 6FXJ) in overlay with H117A (PDB: 9T54). As the WT, the mutant crystallizes as a pentamer with the flexible loop fully resolved in two out of five chains (A and C). The areas with low electron densities confirming the loop have either been cut from the model or show minor differences in fold. The overlay in Fig. 10B highlights a general structural consensus between chain A of the WT and chain A of H117A with a calculated RMSD of 0.166 Å. Figure 10A,C present a detailed look on different noteworthy discrepancies between WT and H117A.

**Table 2:** Data collection parameters, model properties, refinement statistics of the crystal structure from *Lm*ChdC H117A (PDB‐ID: 9T54).

Coproheme decarboxylase H117A mutant from *Listeria monocytogenes* in complex with iron coproporphyrin III	
PDB‐ID	9T54
**Data collection**	
Wavelength [Å]	0.9677
Resolution range [Å]	65.254–2.19 (2.268–2.19)
Space group	P 1 21 1
Unit cell dimensions	*a* = 78.429 Å, *b* = 129.3 Å, *c* = 78.6509 Å; *α* = 90°, *β* = 106.049°, *γ* = 90°
Total reflections nr.	551 673 (56392)
Unique reflections nr.	76 424 (7609)
Multiplicity	7.2 (7.4)
Completeness (%)	98.51 (96.78)
Mean *I*/*σ*(*I*)	6.92 (1.16)
Wilson B‐factor	36.2
*R*_merge_	0.3247 (2.636)
*R*_meas_	0.3498 (2.832)
*R*_pim_	0.129 (1.028)
CC1/2	0.986 (0.405)
**Refinement**	
Reflections used in refinement	76 213
Reflections used for R‐free	1754
*R*_work_	0.1804
*R*_*free*_	0.2061
RMS(bonds)	0.012
RMS(angles)	1.99
Ramachandran favored (%)	97.72
Ramachandran allowed (%)	1.95
Ramachandran outliers (%)	0.33
Rotamer outliers (%)	3.39
Clashscore	6.34
Average B‐factor	44.79
Macromolecules	44.5
Ligands	57.95
Solvent	38.35
**Model properties**	
Number of models	1
Number of chains	6
Number of atoms (non‐H)	10 326
Macromolecules	10 046
Ligands	245
Solvent	35
Protein residues	1240
Overall number of H‐D atoms	0

**Fig. 10:**
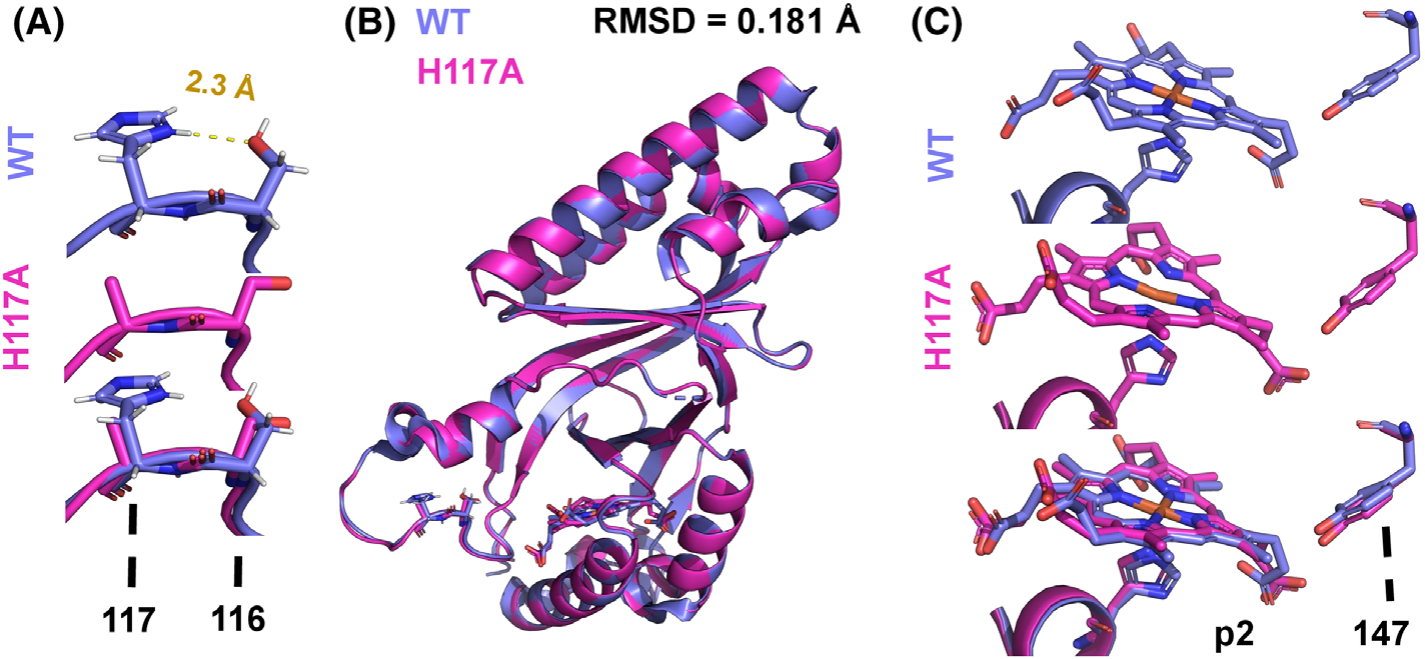
Overlays between WT (blue, PDB‐ID: 6FXJ) and H117A (pink, PDB‐ID: 9T54). Chain A of both proteins was used. Figures were generated in pymol. (A). Overlay of the loop region containing H117/A117 and S116. Hydrogens were added to highlight the polar contact found in 6FXJ. (B). Full subunit overlay. (C). Coproheme molecule with propionate 2 coordination towards conserved, catalytic residue Y147.

Additionally, the macromolecular structure of mutant and WT aligns very well Fig. S4. All subunit alignments between them have an RMSD of < 0.25 Å and, as already mentioned above, they both crystallize as homopentamers with their subunits associated in a ring shape.

In Fig. 10A, the flexible loop is focused: Residues 116 and 117 are presented as sticks on top of the cartoon representation. In the WT structure, a polar contact is predicted between H117 and S116, with S116's hydroxyl group being oriented towards N_δ1_ on H117's side chain. This highlights a possible interaction to maintain conformational stability of this region of the loop. In the mutant S116 changes orientation, since the H117A mutation cannot act as a polar interaction partner for the serine.

This observation is alternatively supported by the MD simulations of the WT: When side chains hydrogen bonds of H117 and S116 are analyzed, the same hydrogen bond occurs with a frequency of 93.31%, while a second hydrogen bond between H117, in inversed position, and S116 (H117, N_ɛ2_ to S116 O_γ_) only occurs with a small frequency of 0.18%.

As already mentioned, the MD simulations presented before are elongations and adapted versions from already published MD results. Hence, they were conducted on modeled *Lm*ChdC structures (PDB‐ID: 4WWS with coproheme modeled into the active site and the H117A mutation introduced in‐model). RMSD calculations between the crystal structures of *Lm*ChdC WT (PDB: 6FXJ) or H117A (PDB: 9T54) between the central clusters of the simulation (Fig. 3C) reveal all five loops of 6FXJ closely match the predominant cluster 1 with an RMSD of < 2 Å. For 9T54, only the fully resolved loops of chain A and C match cluster 1 in the same RMSD threshold. The other loop regions deviate, which seems understandable, given they only have only little electron density to confirm their modeling.

Another notable difference between WT and H117A is found in the active site (Fig. 10C), where p2 (propionate 2) is oriented differently towards Y147, an essential catalytic residue of *Lm*ChdC.

To add validation to those observations, figures from webcoot/moorhen were prepared in Fig. S5. They show different chains of the model fitted into the electron density map and make some degree of qualitative assessment of the fit available to the reader. Figure S5A,B show the highlighted regions from Fig. 10 in chain A, Fig. S5C shows the highlighted region of the active site from chain B, and Fig. S5D shows the active site loop in chain C.

## Discussion

Overall, the results presented indicate that H117 has a stabilizing function for the loop that covers the active site of *Lm*ChdC. In the presented X‐Ray structure (Table 2, Fig. 10) and based on the results of the MD simulations, a polar contact with the neighboring S116 seems to be maintained and keeping the residue in place.

When the H‐bond forming H117 is exchanged to an alanine, the simulations further suggest a greatly increased fluctuation and flexibility of the loop (Fig. 3), which has consequences on the binding of bulky cofactor/substrate coproheme (Fig. 5) and the small iron‐ligand cyanide (Figs 8, 9). Global structural implications can be observed when comparing CD spectra of WT and mutant (Fig. 4).

Coproheme binding happens without the formation of an intermediate state and is monophasic in the H117A variant, while the WT shows biphasic coproheme binding (Fig. 5). In the WT, an intermediate compound is forming during the presteady‐state phase and is probably associated with a substrate reorientation or a conformational change during the binding process. Both can be caused by a more rigid loop covering the active site.

Generally, loop arrangement marks a big difference to actinobacterial ChdCs. As already described in the introduction, these ChdC homologs differ to *Lm*ChdC, with their catalytically conserved histidine residue being coordinated towards the coproheme center by the shorter linker [9, 10].

The binding behavior of cyanide to coproheme seems to be influenced by the mutation as well (Figs 8, 9, Table 1): The multi‐exponential fitting of the time traces for ligand binding to the WT in comparison to the clear single exponential fit of the H117A time traces, as well as faster cyanide binding of this variant at pH 7, indicate the presence of a second, concentration‐independent and binding‐influencing hinderance into the WT active site. This additionally highlights the increased active site accessibility caused by the H117A mutation.

H117A exhibits a significantly higher binding rate for cyanide at pH 7 compared to the WT (Table 1). It would be expected that the binding rates for both the WT and the H117A mutant increase at pH 9, based on the assumption that the chemical equilibrium H^+^ + CN^−^ ⇌ HCN is shifted to the deprotonated form, making cyanide binding faster. This holds true for the WT but interestingly not for H117A. A possible explanation could be that the hydrogen bond between S116 and H117 is disrupted under basic conditions, but the histidine is still mediating an undefined effect improving substrate accessibility, most likely by steric terms.

For actinobacterial *Cd*ChdC, cyanide binding, measured on the same instrument and a highly similar set up, at pH 7 for both *Cd*ChdC WT and the *Cd*ChdC H118A mutant has an increased *K*_D_ of 20–30 μm (WT) and 27–37 μm (H118A) with corresponding lower *k*_on_‐values, compared to *Lm*ChdC WT and H117A [9]. This indicates a shortened loop leads to lower accessibility for cyanide when comparing the different ChdC homologs, regardless of the histidine position. While the histidine‐to‐alanine mutation does not cause a large change in *K*_D_ to cyanide, heme bleaching upon H_2_O_2_ exposure is observed for *Cd*ChdC H118A [9], however to a lesser extent than for *Lm*ChdC H117A.

When analyzing activity results for *Lm*ChdC presented in this study, the clearest conclusion which we can draw is a higher susceptibility to cosubstrate mediated oxidative damage by the cosubstrate to H_2_O_2_, which also inhibits enzyme activity to some degree (Figs 6, 7). This subsequently highlights H117 as a necessary stabilizing entity ‘guarding’ the active site from oxidative damage—a role which H118 in *Cd*ChdC is not required to fulfill to the same extent, given the reduced prevalence of heme bleaching for *Cd*ChdC [9].

Ultimately our observations lead to the conclusion, that the longer loop in *firmicute* ChdCs allows a higher degree of active site access. This access is reduced for actinobacterial ChdCs with their reduced loop length, regardless of the position of their catalytic histidine residue. An interesting side observation is the seemingly higher degree of bleaching, which heme *b* undergoes when compared to coproheme. This is shown by a comparable absorbance loss at 395 nm between WT and H117A and an increased absorbance loss at 410 nm for the mutant (Fig. 7). To our knowledge, the vulnerability to bleaching between different enzyme coordinated iron bound porphyrin species has not been tested yet and might be an interesting starting point for future studies.

The second observation from the X‐ray crystallography study conducted here is a difference in the coordination of propionate 2 for H117A compared to the WT structure. This seems to align with the observed changes in coproheme binding behavior. The presence of an intermediate state in binding has been described before and not only been observed for *Lm*ChdC but also for *Sa*ChdC (*Staphylococcus aureus*). Propionate 2 coordination in *Lm*ChdC WT shows fleeting hydrogen bond formation in molecular dynamics simulation, indicating the exact coordination might not be the deciding factor for enzyme activity [20] but possibly for substrate reorientation [12].

To summarize, the histidine positioned in the loop of *Lm*ChdC does not seem to have a single distinctive effect like the analogous residue in actinobacterial ChdCs. However, mutating it to an alanine and therefore making the loop covering the active site, an already flexible element, even more flexible, apparently causes a loss of cover. Subsequently, protection for the active site of the enzyme is lost. This could highlight a regulatory effect for substrate binding, product release, and finally cosubstrate transfer to coproheme as biological functions to this structural element.

## Materials and methods

### B‐factor analysis of deposited ChdC structures and multiple sequence alignment (MSA)

For the B‐factor analysis presented in the introduction, all ChdC structures found in the PDB were included (September 2025, PDB IDs: 1VDH, 1T0T, 4WWS, 5LOQ, 5T2K, 6FXQ, 6FXJ, 6XUB, 6XUC, 6VSA, 6VSC, 7Q4G, 7Q4F). Only the first chain of the respective model was used. A consensus sequence was generated by clustalw, and the average B‐factors of the respective numerical position in the consensus was plotted.

To identify conserved residues in the protein sequence, a multiple sequence alignment was performed on 32 *Firmicutes* derived ChdC sequences as described by Pfanzagl *et al*., [21]. Loop areas were identified by investigating the crystal structure of 6FXJ and revealed a V/I‐E‐L‐S motif (see Fig. 2) as its conserved starting sequence. This served as an anchor point for depicting the alignment by Berkeley Weblogo, once for all 32 sequences and once for the five *Listeria* sequences in the dataset [22].

### Molecular dynamics simulation (MD) of *Lm*ChdC WT and H117A mutant

The set‐up for molecular dynamics simulation was described in detail [12]. The previously conducted simulations for *Lm*ChdC WT were prolonged to a length of 50 ns, while for the H117A mutant, a local mutation was introduced into the WT starting model and the corresponding simulation was performed. In short, a crystal structure of *Lm*ChdC WT (PDB ID: 4WWS) was used, missing flexible loops were reconstructed and coproheme was merged into the five active sites. Simulations proceeded in GROMOS11, applying the 54a8 forcefield [23]. After energy minimization in vacuum, proteins were solvated in a rectangular box containing periodic simple point charge water molecules and minimum solute to wall distances set to 0.75 nm in all dimensions [24]. The potential energy was minimized in water, and 65 sodium atoms were added to ensure overall neutrality. Initial velocities were assigned according to a Maxwell–Boltzmann distribution at 60 K for equilibration, solute atoms were positionally restrained and in four short simulations the force constant for the positional restraints was reduced by one order of magnitude, while the temperature was increased by 60 K. Finally, position restraints for the solutes were removed, roto‐translational constraints were introduced and a last step of equilibration was performed at 1 atm for 300 ps [25]. Equilibrium simulations were run with a constant number of particles, constant temperature (300 K) and constant pressure (1 atm).

While original runs resulted in 15–35 ns runtime, the simulations presented in this study were extended to 50 ns.

Since ChdC structures are pentameric, all analyses were performed separately for all monomers. The RMSF (root mean square fluctuation) was computed individually for each loop (residues 113–134, 221 backbone atoms per loop). Hydrogen bond occurrence was determined using a hydrogen‐acceptor distance cut‐off of 0.25 nm and a donor‐hydrogen‐acceptor angle cut‐off of 135°. The occurrences for the loop area were collected and averaged over the five monomers. Conformational clustering based on the RMSD of the backbone atoms of the loop regions (after a least‐square roto‐translational fit on the backbone of the entire monomer) was performed using a cut‐off of 0.20 nm, using the same definition of the loop backbone atoms.

### Cloning and mutagenesis of *Lm*ChdC WT and H117A

A previously described vector served as a template for the *Lm*ChdC insert [12]. It was amplified and inserted by Gibson Assembly (NEBuilder® HiFi DNA Assembly by New England Biolabs GmbH, Frankfurt, Germany) into a modified pETM11 vector, merging the target to a 6× His tag, interspersed by a cleavage site for HRV3C precision protease. The H117A mutation was introduced into the wild‐type (WT) by site‐directed mutagenesis (QuikChange Lightning Kit by Agilent). Finally, the plasmids were transformed into *Escherichia coli* BL21 *Gold* for recombinant expression. Open reading frames with constructs are shown in Table S1.

### Recombinant expression, reconstitution, and purification in *Escherichia coli*

The cells were incubated at 37 °C until an OD_600_ of about 0.6 was reached. Then, the culture was induced by the addition of 0.1 mm IPTG to the cell suspension and further incubated at 18 °C overnight. The cells were harvested by centrifugation at 4 °C, 3170 ***g*** for 20 min.

Purification proceeded with the pellets being suspended in lysis buffer (50 mm HEPES, 500 mm NaCl, 5% glycerol, 0.5% Triton X‐100, pH 7.4). Cell lysis was conducted by sonication on ice (90% amplitude, 3 × 1 min), and the supernatant was filtered and applied onto a *HisTrap* column (Cytiva, Linz, Austria). To elute the protein, the column with target protein bound was equilibrated with cleavage buffer (Tris/HCl 50 mm, NaCl 150 mm, EDTA 1 mm, pH 7.0); then, a surplus of pure HRV3C precision protease was applied onto the column, and it was incubated at 4 °C overnight. Elution proceeded on the next day with 15 mL of storage buffer (50 mm HEPES, 200 mm NaCl, pH 7). The cleaved product was reconstituted by adding a molar surplus of coproheme (Fe‐coproporphyrin III from Frontier Scientific, Logan, Utah, USA), dissolved in 10 mm NaOH. To remove excess coproheme and remaining impurities, the sample was concentrated and applied to a size exclusion column (120 mL HiLoadTM 16/600 SuperdexTM 200 pg column by Cytiva, Linz, Austria). Target protein eluted with a strong peak visible at 280 and 390 nm. The fractions were concentrated and stored at −80 °C.

### High‐pressure liquid chromatography (HPLC) for size determination and quality control

To confirm quality, 50 μg of protein was injected on a size exclusion column (Superdex 200 10/300 GL, particle size 13 μm by GE Healthcare, Vienna, Austria) by a high‐pressure liquid chromatography system (HPLC) (Shimadzu Prominence LC20 by Shimadzu Europe GmbH, Korneuburg, Austria). For detection, a multi‐angle light scattering (MALS) device (Heleos Dawn8+ by Wyatt Technology Corporation, Dernbach, Germany) and a diode array detector (SPD‐M20A by Shimadzu Europe GmbH, Korneuburg, Austria) were in place. The mobile phase consisted of *Dulbecco phosphate‐buffered saline* (PBS) with 200 mm NaCl. Results for the quality check are depicted in Fig. S1.

### Comparative circular dichroism (CD) spectroscopy

Roughly 10 μm of apo‐*Lm*ChdC WT and H117A were prepared in low‐salt buffer (5 mm phosphate buffer, pH 7) and adjusted to an equal UV–vis absorbance at 280 nm. Measurements were performed with a ChirascanTM VX (Applied Photophysics Ltd., Leatherhead, UK) in a UV wavelength range of 190–240 nm.

### Instrumental details for UV–vis spectroscopy

Conventional UV–vis spectroscopy was conducted with a Cary60 instrument (Agilent). By default, spectra were recorded between 250 and 700 nm and a scan speed of 720 nm·min^−1^. The samples were prepared in a volume of 1 mL in a continuous stirred quartz cuvette.

Stopped‐flow spectroscopy was performed with two instruments: a *PiStar‐180* and a *SX18‐MV*, the former equipped with a monochromator and photomultiplier, and the latter with a photodiode array detector. Both instruments were acquired from Applied Photophysics Ltd.

### Coproheme binding to apo‐ *Lm*ChdC measured by stopped‐flow spectroscopy

About 2 μm of dissolved coproheme was mixed with 6 μm of apo‐*Lm*ChdC WT or H117A by the *SX18‐MV*. Spectral changes were recorded by a photodiode array over 5 s with 500 spectra in logarithmic frequency to record rapid binding. Fitting the data and identifying intermediate species and their molar ratios was done with Pro‐Kineticist (Applied Photophysics Ltd) as already shown [20].

### Cyanide binding to holo‐ *Lm*ChdC in steady state

*K*_D_ values toward cyanide were determined by titrating a 5 μm protein sample in 50 mm phosphate or borate buffer set to pH 7 and 9 with cyanide dissolved in the respective buffer. Spectra were taken at increasing ligand concentrations using UV–vis spectroscopy as described above.

*K*_D_ calculation from the titration data proceeded by subtracting the reference spectrum from each spectrum at different cyanide concentrations. With incrementing concentrations of cyanide, the difference in absorption to the original spectrum at the respective Soret maxima rose as well, until saturation was reached. In a graph, in which those absorption differences were plotted against the cyanide concentration, the data follows a hyperbolic function, fitted with the equation:

$${\text{ΔAbs}}_{\left(p-v\right)}=\frac{{\text{ΔAbs}}_{\left(p-v\right)\;\max}*\left[{\mathrm{CN}}^{-}\right]}{K_{D}+\left[{\mathrm{CN}}^{-}\right]}$$

ΔAbs_(p–v)_ represents the sum of the difference of absorbance at the respective absorbance minimum at 380 nm (v for valley) and maximum at 415 nm (p for peak) for each individual cyanide concentration. ΔAbs_(p–v) max_ is the maximal difference, which is reached when saturation with ligand is achieved. *K*_D_ was finally determined by fitting the data *with* sigmaplot *14.0* (systat).

### Cyanide binding to holo‐ *Lm*ChdC in presteady‐state

Cyanide binding in presteady‐state was measured with the *PiStar‐180* stopped flow system at a fixed wavelength of 412 nm. Final concentrations of 2 μm of protein reconstituted by coproheme were incubated with 50–600 μm of dissolved cyanide. Each sample was measured in triplicate. *k*_obs_‐values were determined by applying different exponential fits in Pro Data Viewer SX (Applied Photophysics Ltd), which were then plotted against cyanide concentration and fitted by linear regression, from which the *k*_on_ was determined as the slope. The number of exponential components in the fit was decided by overlaying experimental and fitted data. Single exponential fitting followed according to the following models (a) single exponential fit, (b) double exponential fit, (c): triple exponential fit:

(a) $$\;y=a_{1}*e^{-k_{\mathrm{obs},1}*x}+c$$

(b) $$\;y=a_{1}*e^{-k_{\mathrm{obs},1}*x}+a_{2}*e^{-k_{\mathrm{obs},2}*x}+c$$

(c) $$\;y=a_{1}*e^{-k_{\mathrm{obs},1}*x}+a_{2}*e^{-k_{\mathrm{obs},2}*x}+a_{3}*e^{-k_{\mathrm{obs},3}*x}+c$$

*k*_off_ was calculated using *K*_D_ from the titration experiment and *k*_on_ from the presteady‐state experiment.

### LC–MS for relative quantification of porphyrins

The method for the relative quantification of the relevant porphyrins was established by Michlits *et al*., [9] and adapted accordingly. In short, after injection the sample was separated over a 9‐min gradient (15% acetonitrile in 0.1% formic acid → 80%). The flow rate was set to 0.4 mL·min^−1^. The column was a *Waters BioResolve* column (2.1 × 5 mm), heated to 80 °C in an Agilent 1290 Infinity II UPLC system. For detection, a time‐of‐flight MS (Agilent Series 6230 LC‐TOFMS by Agilent) was used in positive ion mode.

### X‐ray crystallography on *Lm*ChdC H117A

Crystallographic screening was performed with coproheme reconstituted *Lm*ChdC H117A, (stored at 200 μm in 50 mm phosphate buffer, pH 7, 200 mm NaCl), preincubated with cyanide to prevent unwanted substrate conversion. Plate‐like crystals were growing by vapor diffusion after 2–3 days in a sitting drop containing 0.25 μL protein solution (5 g·L^−1^ protein, 1 mm cyanide, prepared in 70 μL, diluted with HQ‐H_2_O) mixed with 0.2 μL of mother liquor (0.2 m ammonium iodide, 20% w/v PEG 3350 in HQ‐H_2_O). Plates were set up using a Mosquito LCP pipetting robot (TTP Labtech, Melbourn Science Park, Melbourn, UK). Crystals were fished with 25% of glycerol as a cryoprotectant, stored in liquid N_2_ and shipped to ESRF Grenoble, FR (European Synchrotron Radiation Facility), where they were shot at the ID30A3 (MASSIF‐3) beamline (https://doi.esrf.fr/10.15151/ESRF‐ES‐2210529088) [26].

Full processing was performed with the available XIA2 automated pipeline [27, 28]. Afterwards, molecular replacement and refinement were done in ccp4 cloud v.1.8. 009 [29]. Molecular replacement was done by Phaser 2.8.3 with an AlphaFold prediction as an input model [30]. B‐factors were recalculated by Slice‐n‐Dice [31]. Finally, refinement was conducted by Refmacat with model building by coot and moorhen/webcoot [32, 33, 34, 35, 36, 37].

The dataset showed pseudo‐merohedral twinning, which was considered during the refinement process with twinning operator ‐L, ‐K, ‐H and with a twinning fraction of 0.14 refined during final cycles, consistent with the *L*‐test. Further a resolution cut‐off was set to 2.2 Å. Overall refinement led to a suitable structure. Very flexible elements (linking loops, N‐termini), where no electron densities supported the model were cut.

The structure was deposited under PDB‐ID: 9T54.

## Conflict of interest

The authors declare no conflict of interest.

## Author contributions

NF and SH planned experiments; NF and DH performed experiments; NF, DH, CO, PGF, and SH analyzed data; NF, DH, CO, PGF, and SH wrote the paper.

## Supporting information


**Table S1:** Sequence information of *Lm*ChdC WT and H117A as expressed in this study.
**Fig. S1:** Backbone H‐bonds/polar contacts between a residue's N–H as donor and another residue's O/OXT as acceptor.
**Fig. S2:** HPLC chromatograms of WT (blue) and H117A (violet).
**Fig. S3:** Titrations of coproheme with WT and H117A.
**Fig. S4:** Overlays between WT (blue, PDB‐ID: 6FXJ) and H117A (pink, PDB‐ID: 9T54) prepared in pymol.
**Fig. S5:** Electron density analysis of different areas of the H117A structure (PDB‐ID: 9T54) relevant to the study.

## Data Availability

The data collected in this study is deposited in the zenodo repository and can be accessed via this doi: https://doi.org/10.5281/zenodo.19663585.
