# Supplementary material for: Unraveling the active site cover of coproheme decarboxylase from Listeria monocytogenes
Source: FEBS J. 2026 May 6;293(20):6272–88. doi: 10.1111/febs.70575 (PMC13669642; doi:10.1111/febs.70575)
Supplement: Supplementary file 1 [file FEBS-293-6272-s001.pdf]

Supporting Information:

Unravelling the active site cover of coproheme decarboxylase from *Listeria monocytogenes*

Nikolaus Falb[1], Dominik Hörtnagel[2], Chris Oostenbrink[2], Paul G. Furtmüller[1], Stefan Hofbauer[1]*

[1] BOKU University, Institute of Biochemistry, Department of Natural Sciences and Sustainable Resources, Vienna, Austria

[2] BOKU University, Institute of Molecular Modeling and Simulation, Department of Natural Sciences and Sustainable Resources, Vienna, Austria

***** corresponding author, e-mail: stefan.hofbauer@boku.ac.at

Table S1: Sequence information of *Lm*ChdC WT and H117A as expressed in this study. 6×His-Tag and sequence removed by protease are underscored.

***Lm*ChdC WT in pETM11**
MKHHHHHHGLEVLFQGPAMNEAVKTLDGWFCLHDFRSIDWAAWRELNPGNQELMLNEL<br>SHFLSDMEITKNIGEGEHTIYSILGQKADLVFFTLRDSLEALNEVENRFNKLAIADYLLPTYS<br>YISVVELSNYLAS**H**MAGGDDPYQNKGVRARLYPALPPKKHICFYPMSKKRDGADNWYML<br>PMEERQQLIRDHGLIGRSYAGKVQQIIGGSIGFDDYEWGVTLFSDDALEFKRIVTEMRFDE<br>ASARYAEFGSFFIGNLLLSEQLSKLFTI*
atgaaacatcaccaccaccaccatggactggaagttctgttccaggggcccgccatgaatgaagcagtaaaaacgttagac<br>ggttggttttgtttacatgattttcgttcgattgattgggctgcatggcgtgaattaaatccaggtaatcaggaacttatgttaaatgaa<br>ctaagccattttttaagcgatatggaaattactaaaaatatcggtgaaggagaacataccatttacagcattcttggtcaaaaag<br>cagatttagtattcttcactttgcgtgattcccttgaagcgctaaatgaagtcgaaaatcgttttaataaattagcgattgccgattac<br>ttattaccaacttattcctatatatctgtggtggaactgagcaactatcttgcgtct**cac**atggctggcggtgacgatccttatcaaa<br>ataaaggtgttcgcgcgagactctatcctgcacttccgcctaaaaagcatatttgtttctacccaatgagtaaaaaaagggatgg<br>cgctgataattggtacatgcttccaatggaagaacgccaacaacttattcgcgaccacggcttgattggcagaagctatgctgg<br>caaagtacaacaaatcattggtggctccattggatttgatgattatgaatggggcgtcactttgttctctgatgatgcactggagttc<br>aaacgcatcgtaacggaaatgcgctttgatgaagcaagtgctcgttacgccgaatttggctctttctttattggaaacctgttactat<br>ccgagcaactttctaaattatttactatttag
***Lm*ChdC H117A in pETM11**
MKHHHHHHGLEVLFQGPAMNEAVKTLDGWFCLHDFRSIDWAAWRELNPGNQELMLNEL<br>SHFLSDMEITKNIGEGEHTIYSILGQKADLVFFTLRDSLEALNEVENRFNKLAIADYLLPTYS<br>YISVVELSNYLAS**A**MAGGDDPYQNKGVRARLYPALPPKKHICFYPMSKKRDGADNWYML<br>PMEERQQLIRDHGLIGRSYAGKVQQIIGGSIGFDDYEWGVTLFSDDALEFKRIVTEMRFDE<br>ASARYAEFGSFFIGNLLLSEQLSKLFTI*
atgaaacatcaccaccaccaccatggactggaagttctgttccaggggcccgccatgaatgaagcagtaaaaacgttagac<br>ggttggttttgtttacatgattttcgttcgattgattgggctgcatggcgtgaattaaatccaggtaatcaggaacttatgttaaatgaa<br>ctaagccattttttaagcgatatggaaattactaaaaatatcggtgaaggagaacataccatttacagcattcttggtcaaaaag<br>cagatttagtattcttcactttgcgtgattcccttgaagcgctaaatgaagtcgaaaatcgttttaataaattagcgattgccgattac<br>ttattaccaacttattcctatatatctgtggtggaactgagcaactatcttgcgtct**gcc**atggctggcggtgacgatccttatcaaa<br>ataaaggtgttcgcgcgagactctatcctgcacttccgcctaaaaagcatatttgtttctacccaatgagtaaaaaaagggatgg<br>cgctgataattggtacatgcttccaatggaagaacgccaacaacttattcgcgaccacggcttgattggcagaagctatgctgg<br>caaagtacaacaaatcattggtggctccattggatttgatgattatgaatggggcgtcactttgttctctgatgatgcactggagttc<br>aaacgcatcgtaacggaaatgcgctttgatgaagcaagtgctcgttacgccgaatttggctctttctttattggaaacctgttactat<br>ccgagcaactttctaaattatttactatttag

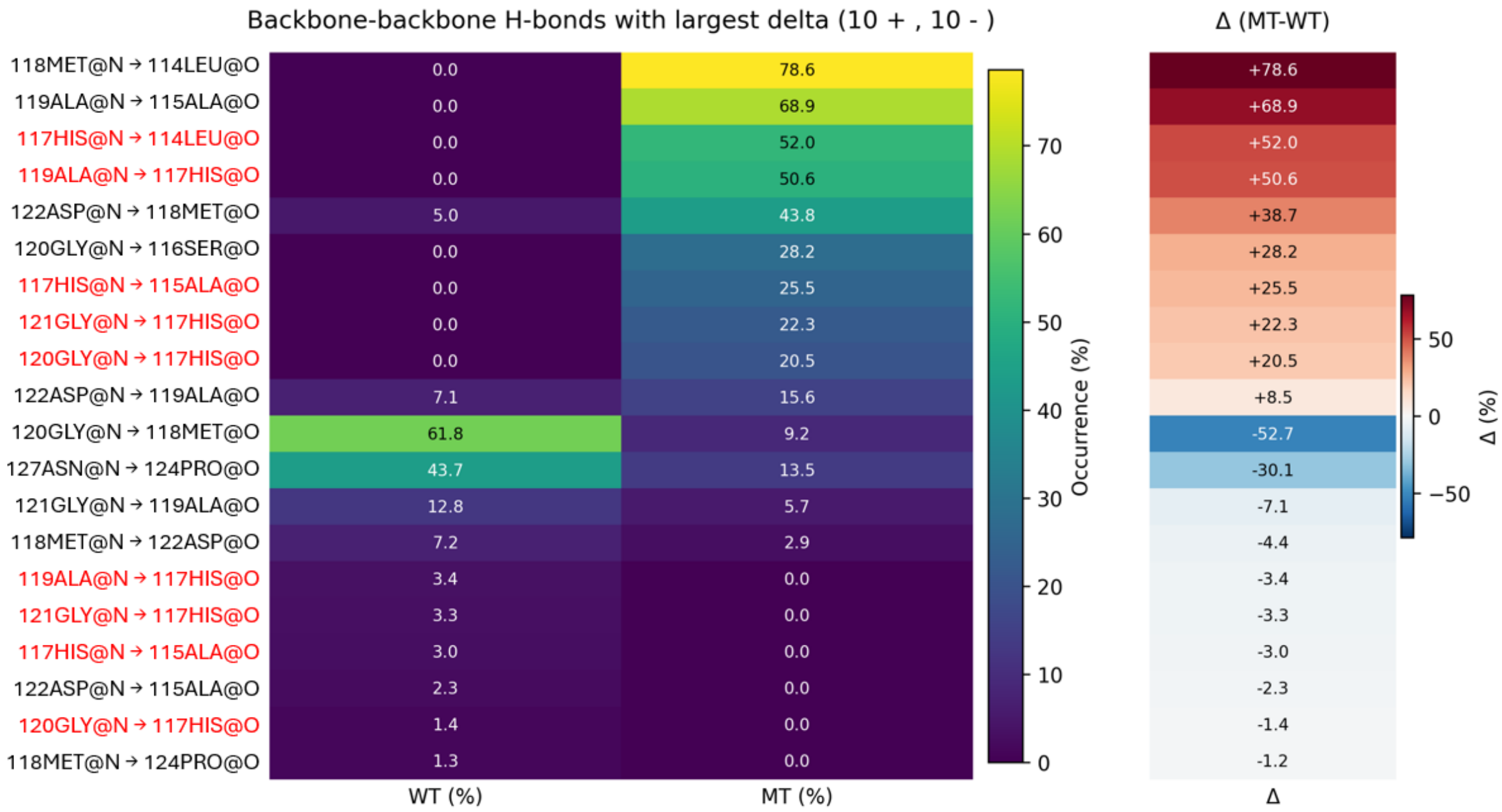

Figure S1: Backbone H-bonds/polar contacts between a residue's N-H as donor and another residue's O/OXT as acceptor. Plot shows H-bonds with largest differences in occurrence (%) between WT and mutant MT (H117A). 10 H-bonds with largest positive difference and 10 with largest negative difference are presented with their original occurrences and their difference Δ (MT-WT).

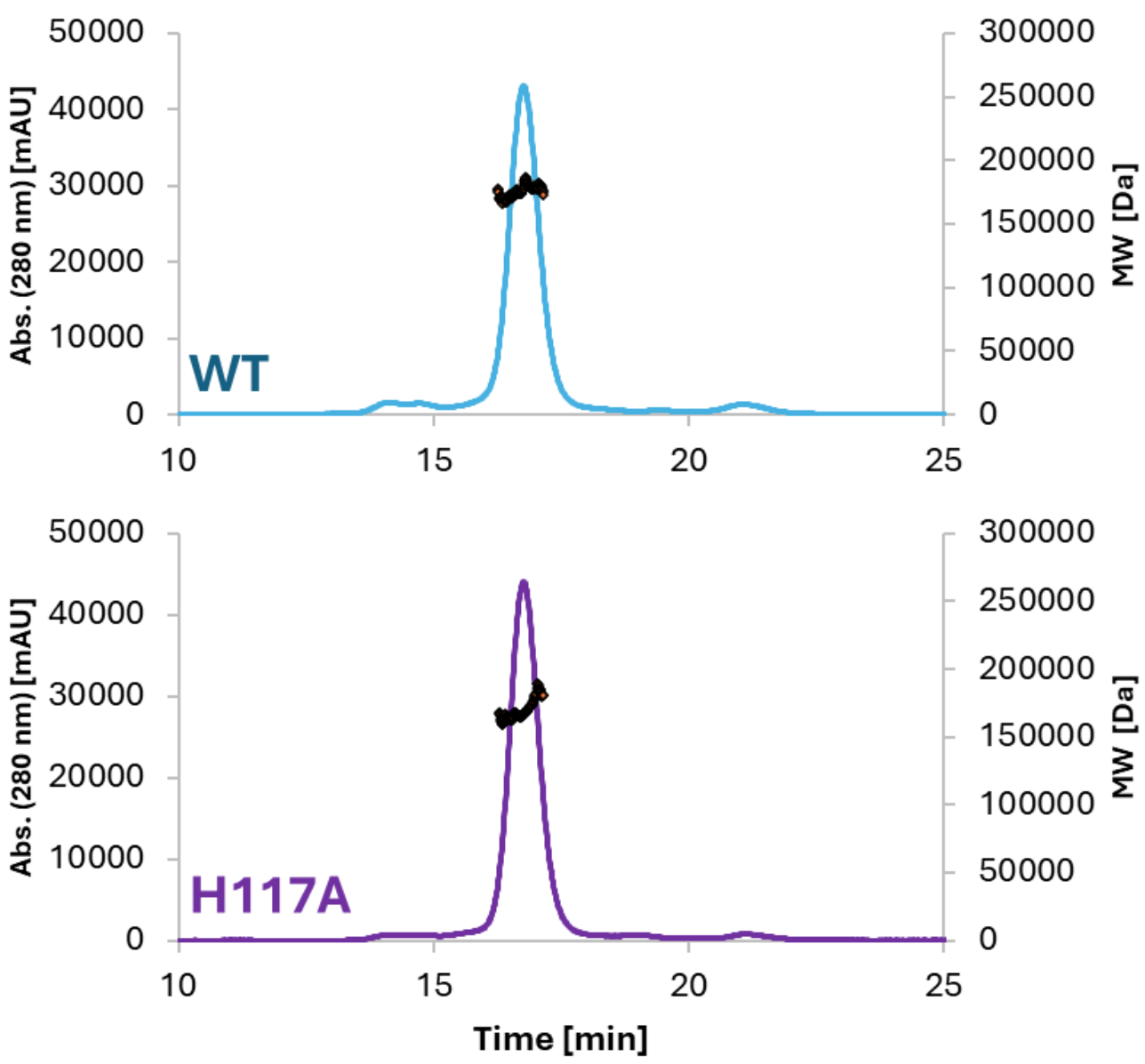

Figure S2: HPLC chromatograms of WT (blue) and H117A (violet). Coloured line shows the detected absorbance at 280 nm. Dots at the peak area indicate MALS signal computed to Dalton.

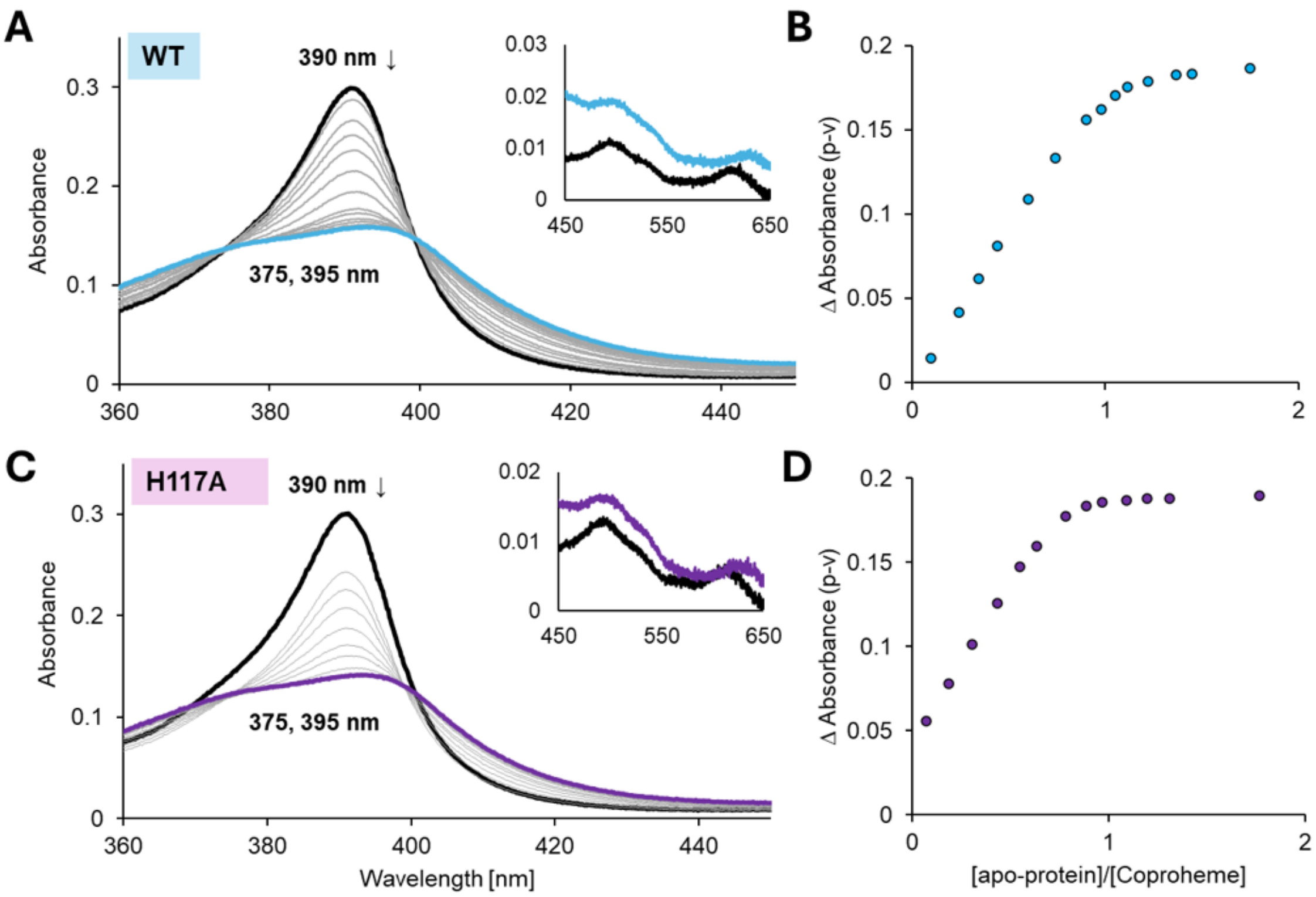

Figure S3: Titrations of coproheme with WT and H117A. **A-B.** Show representative spectral shift of coproheme with rising WT concentration. **C-D.** Show representative spectral shift of coproheme with rising H117A concentration. On the x-axis the molar relationship between apo-protein and coproheme are presented and on the y-axis the summed differences in absorbance at the two wavelengths with largest change to the spectrum of unbound coproheme. "p" refers to a wavelength of 390 nm and "v" refers to a wavelength of 410 nm.

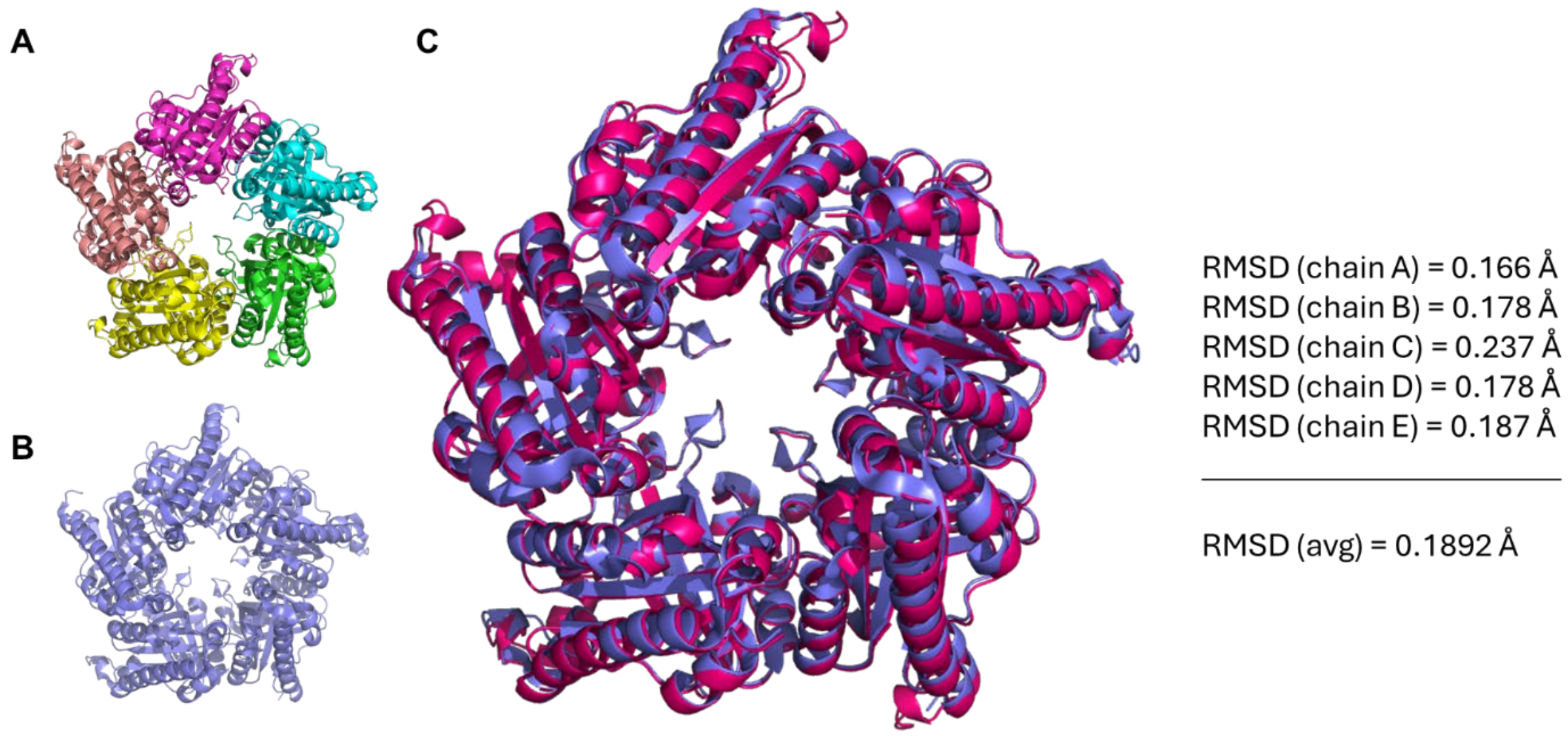

Figure S4: Overlays between WT (blue, PDB-ID: 6FXJ) and H117A (pink, PDB-ID: 9T54) prepared in *PyMol*. **A.** H117A in full pentameric form with subunit chains of the assembly highlighted in different colours. **B.** WT pentamer. **C.** Overlay of both pentamers, based on a Chain A alignment. RMSD-values of aligning different chains to their respective counterpart in the other structure are depicted on the right, including an average.

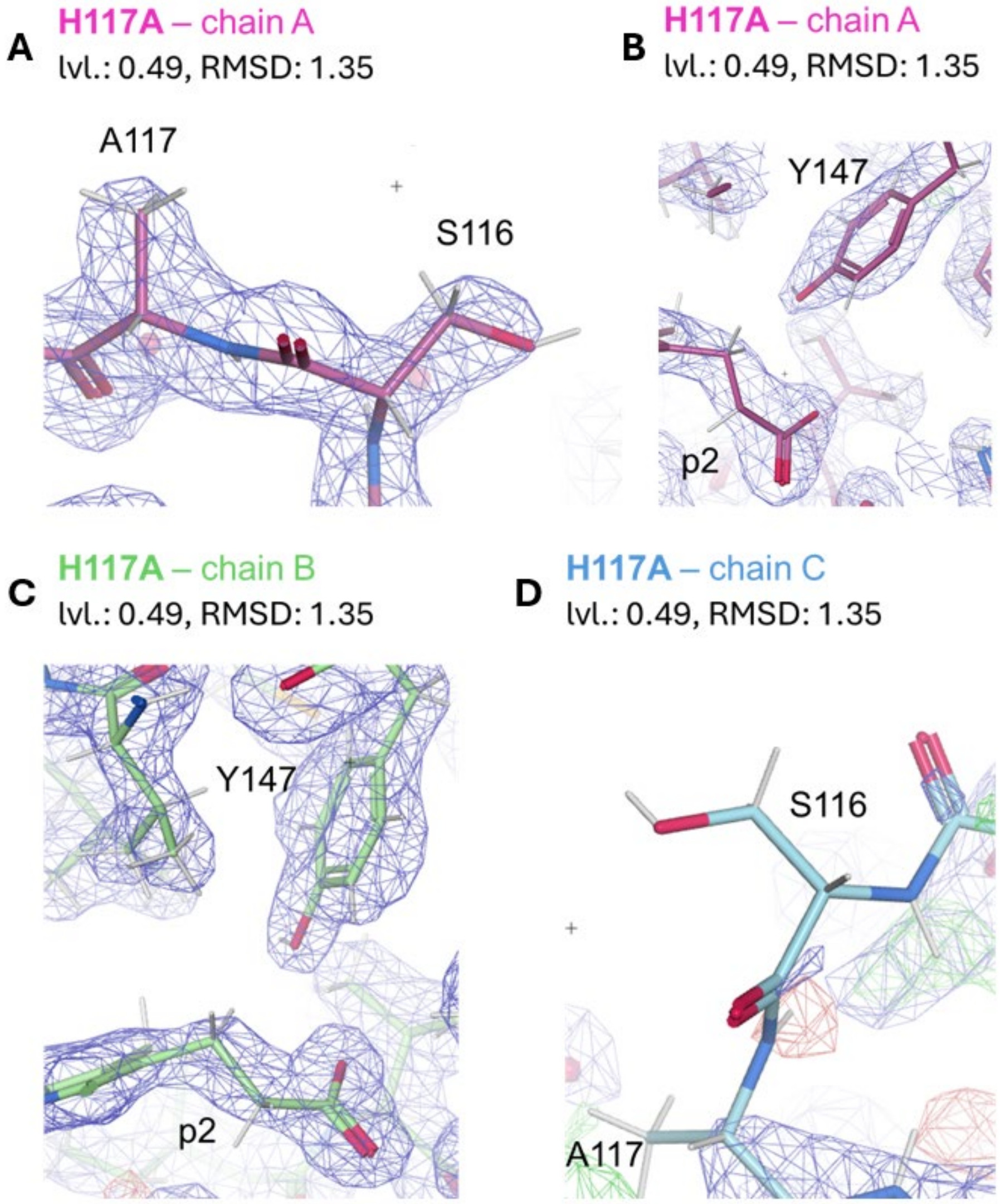

Figure S5: Electron density analysis of different areas of the H117A structure (PDB-ID: 9T54) relevant to the study. Map levels and RMSD values are stated, screenshots were taken in *Moorhen*/*WebCoot.* Electron densities highlighted here fit nicely across different chains. **A.** shows S116 and A117 in the loop region of chain A, **B.** shows Y147 coordinated to propionate 2 of coproheme in chain A, **C.** shows Y147 coordinated to propionate 2 of coproheme in chain B and **D.** shows S116 and A117 in the loop region of chain D.
